# Programming Bacteria With Light—Sensors and Applications in Synthetic Biology

**DOI:** 10.3389/fmicb.2018.02692

**Published:** 2018-11-08

**Authors:** Zedao Liu, Jizhong Zhang, Jiao Jin, Zilong Geng, Qingsheng Qi, Quanfeng Liang

**Affiliations:** State Key Laboratory of Microbial Technology, Shandong University, Jinan, China

**Keywords:** light-sensors, optogenetics, genetic circuits, synthetic biology, feedback control

## Abstract

Photo-receptors are widely present in both prokaryotic and eukaryotic cells, which serves as the foundation of tuning cell behaviors with light. While practices in eukaryotic cells have been relatively established, trials in bacterial cells have only been emerging in the past few years. A number of light sensors have been engineered in bacteria cells and most of them fall into the categories of two-component and one-component systems. Such a sensor toolbox has enabled practices in controlling synthetic circuits at the level of transcription and protein activity which is a major topic in synthetic biology, according to the central dogma. Additionally, engineered light sensors and practices of tuning synthetic circuits have served as a foundation for achieving light based real-time feedback control. Here, we review programming bacteria cells with light, introducing engineered light sensors in bacteria and their applications, including tuning synthetic circuits and achieving feedback controls over microbial cell culture.

## Introduction

Synthetic biology aims to rationally design cell functions. A major aspect of synthetic biology is to explore natural and engineer new parts to be assembled into biological circuits for programmed cell behaviors (Khalil and Collins, 2010; Way et al., 2014; Patil and Dhar, 2015). Programmed biological circuits require signal input and a major way is to use chemical inducers. However, chemical inductions are potentially toxic, have time delay in transport, and are usually irreversible, which restricts its application in dynamic control of cell behaviors. In contrast, light is minimally invasive, fast delivered in high resolution (Renicke and Taxis, 2016; Fernandez-Rodriguez et al., 2017), and shows satisfying reversibility (Motta-Mena et al., 2014; Kawano et al., 2015), which has provided new strategies for dynamic control of cellular activities. While studies on controlling mammalian cells with light have exploded during the past 15 years (Levskaya et al., 2009; Wu et al., 2009; Bacchus and Fussenegger, 2012; Müller and Weber, 2013; Repina et al., 2017; Rost et al., 2017), light's potential in bacteria cells are underexplored.

Here, we review recent advances in programming bacteria with light. We first introduce the evolution of light sensors and how these natural light sensors are further engineered into two-component and one-component systems with distinct signaling properties. Then we introduce applications of these sensors, introducing light in tuning bacterial synthetic circuits at the level of transcription and protein activity control (Table 1). We also introduce light as a novel signal in bridging the gap between cultured microbes and hardware to achieve real-time feedback control of cell behaviors. Most of the achievements introduced here were made in *Escherichia coli*. Mammalian optogenetics are not within the scope of the article and are only occasionally mentioned as proof of concepts.

**Table 1:** Summary of optogenetic system characteristics.

**Optogenetic system**	**Origin**	**Cofactor/Chromophore**	**Wavelength** **λ on/λoff (nm)**	**Time of activation**	**Size (AA)**	**Mechanism**	**Application**	**References**
Cph8/OmpR	Phytochrome	PCB	650/705	Minutes	650/705	Binding P_ompC_	Transcription regulation	Tabor et al., 2011 Levskaya et al., 2005
CcaS/CcaR	CBCRs	PCB	535/672	–	535/672	Binding P_cpcG2_	Transcription regulation	Hirose et al., 2008 Tabor et al., 2011
UirS/UirR	CBCRs	PVB	405/534	–	405/534	Binding P_csiR1_	Transcription regulation	Ramakrishnan and Tabor, 2016
YF1/FixJ	LOV	FMN	430/dark	Seconds	430/dark	Binding P_FixK2_	Transcription regulation	Möglich et al., 2009
BphP1/PpsR2	Phytochrome	BV	760/640	Seconds	760/640	Binding P_Br_crtE_	Transcription regulation	Ong et al., 2018
AsLOV2	LOV	FMN	450/dark	Seconds	143	Caging	Protein interaction/deactivation	Wu et al., 2009
EL222	LOV	FMN	450/dark	Seconds	222	Homodimerization	Transcription regulation	Zoltowski et al., 2013
VVD	LOV	FMN or FAD	450/dark	Seconds	150	Homodimerization	Protein interaction/Subcellular location	Wang et al., 2012
Magnets	LOV	FAD	450/dark	Seconds	nMag:152 pMag:150	Heteodimerization	Protein interaction/Subcellular location	Kawano et al., 2015
Cry2/CIB1	Cytochromes	FAD	450/dark	Second	498	Heteodimerization; Oligomorization	Protein interaction/Subcellular location	Repina et al., 2017
phyB/PIF	Phytochrome	PCB	660/740	Milliseconds	908/100	Dissociation; Heteodimerization	Protein interaction/Subcellular location/signaling	Levskaya et al., 2009
Dronpa	Fluorescent protein	–	500/400	Seconds	224	Oligomorization	Protein interaction/signaling	Zhou et al., 2012 Lv et al., 2017

## Light sensors in bacteria

### Evolution and classification of light sensors

A variety of bacterial and eukaryotic light sensors have evolved to sense ultraviolet, blue, green, red, and near-infrared signals (Purcell and Crosson, 2008; Schmidt and Cho, 2015; Repina et al., 2017; OptoBase, 2018) and have been heterologously expressed in *E. coli*. From an evolutionary prospective, most of the well-tested light sensors in *E. coli* fall into the categories of phytochromes and Light-oxygen-voltage (LOV) family proteins.

Phytochromes share a photosensory core including a PAS (Per-Arnt-Sim) domain, a GAF (cGMP-specific phosphodiesterases, Adenylyl cyclases, and FhlA) domain, and a PHY (Phytochrome-specific GAF-related) domain. The core architecture can be linked to functional domains like Histidine kinase (HK) to become transmembrane sensors of two component systems. The PHY domain incorporates a bilin chromophore to sense red light by photoisomerization (Rockwell and Lagarias, 2010; Burgie and Vierstra, 2014). Cyanobacteriachromes (CBCRs) and bacteriaphytochromes (BphPs) are distant relatives of phytochromes and share a broader range of wavelength sensitivities than phytochromes (Bhoo et al., 2001; Rockwell and Lagarias, 2010; Burgie and Vierstra, 2014). The bilin chromophores required for phytochrome and CBCR/BphP signaling are not present in *E. coli* and have to be synthesized from heme by introducing two genes (Gambetta and Lagarias, 2001).

LOV domains are a member of the PAS domain superfamily and are joint to domains like HK and Helix-turn-helix (HTH) DNA binding domains evolutionally. A variety of methods including light-induced uncaging, tiltering, and dimerization are adopted to control the fused functional domains (Herrou and Crosson, 2011). Like phytochromes, a flavin cofactor (FAD or FMN) is also required for photosignaling of LOV domains but these cofactors are ubiquitous and does not require additional synthesis (Christie et al., 1999) (Table 1).

From an evolutionary view point, both families of light sensors demonstrate great diversity in protein swapping and are naturally joint to different functional modules to become diversified membrane bound HK (two-component system) or cytosolic actuators (one-component system) (Losi and Gärtner, 2008). The understanding of how light sensors evolved into such diversities has inspired human engineering of natural light sensors. Based on their potentials for domain swapping, the light-signaling cores of phytochrome and LOV family proteins are joint to different functional modules artificially into two-component and one-component systems in addition to their natural counterparts. Here, we review engineered light sensors in *E. coli* in the order of two-component and one-component systems according to their distinct signaling properties (Table 1).

### Two-component systems

**Figure 1:**
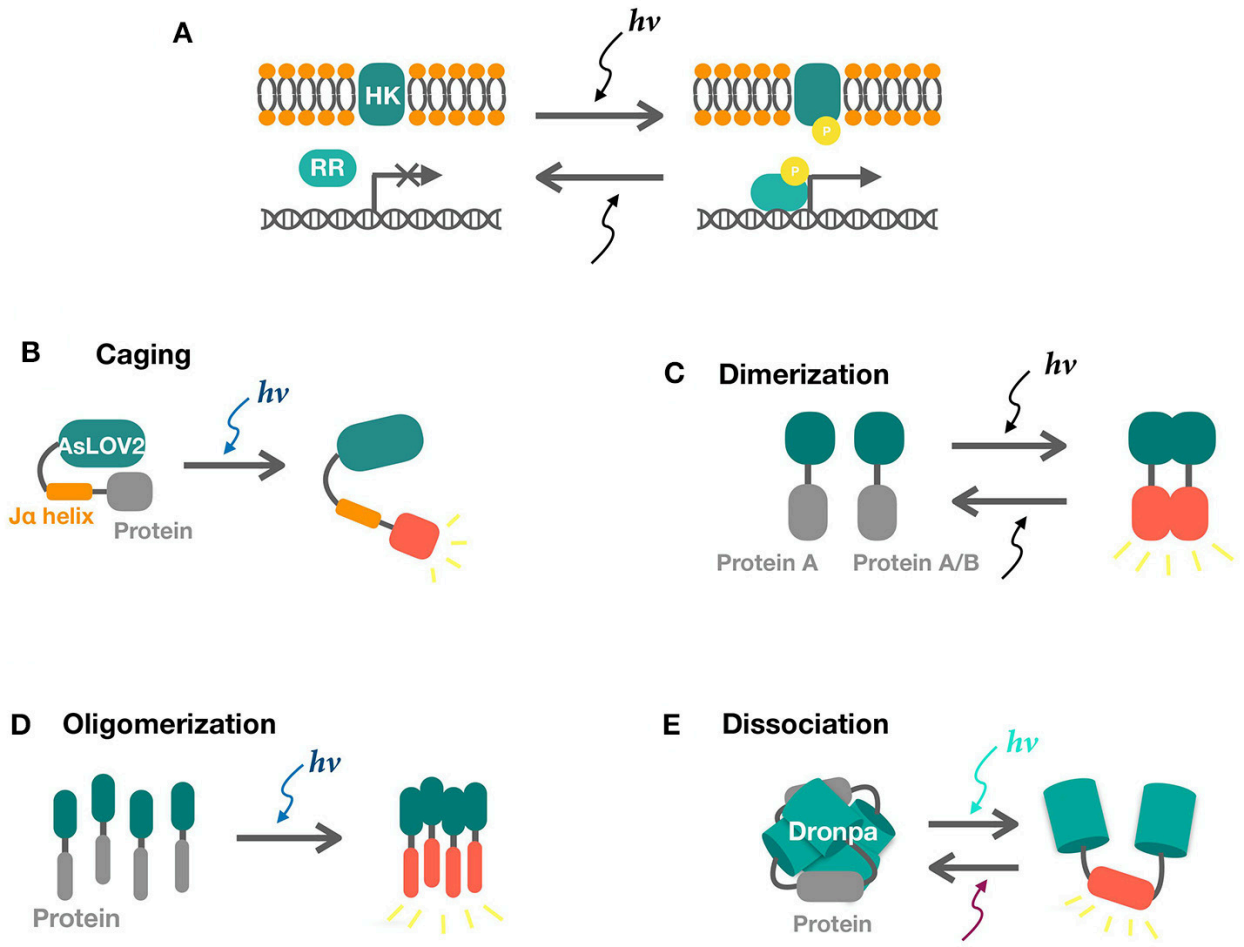
Schematics of two-component and one-component systems. **(A)** Two component systems consist of a sensor histidine kinase (HK) and a response regulator (RR). Activities of response regulators for transcription are tuned by phosphate signaling upon light illumination. **(B)** Photo-induced LOV2-Jα dissociation uncages the fused protein in response to blue light, releasing its activity. **(C,D)** Light-induced dimerization and oligomerization of sensors result in the interaction of the attached proteins. **(E)** Photo-induced dissociation of Dronpa tetramer releases the protein of interest.

The two-component light responding systems (TCSs) have been engineered in bacteria to sense ultraviolet (UirS/UirR) (Ramakrishnan and Tabor, 2016), blue (YF1/FixJ) (Möglich et al., 2009), green(CcaS/CcaR)(Hirose et al., 2008; Tabor et al., 2011), red (Cph8/OmpR) (Levskaya et al., 2005), and near-infrared light (Ong et al., 2018). Light illumination leads to photoisomerization of the cofactor bound HK, tuning kinase activation and inactivation. Signal was further relayed by a phosphate group to the intracellular cognate responsor which controls gene expression under the matched promoter (Figure 1A).

The Cph8/OmpR system was the first engineered two-component system which is switched off by red light. A cyanobacteria phytochrome was used to replace the extracellular signal sensing domain of the original EnvZ/OmpR system (Levskaya et al., 2005). The blue light inactivated YF1/FixJ system was later engineered by swapping the LOV domain in the *Bacillus subtilis* YtvA (Ávila-Pérez et al., 2006) with the original FixL/FixJ system (Möglich et al., 2009). The green light activated CcaS/CcaR system exists naturally in cyanobacteria (Hirose et al., 2008) and have been expressed in *E. coli* (Tabor et al., 2011) and cyanobacteria (Abe et al., 2014; Miyake et al., 2014). Similarly, the UV-light activated UirS/UirR system was also derived from cyanobacteria (Song and Park, 2011).

In contrast to other TCSs, BphP1 is cytosolic and uses light regulated dimerization instead of phosphate signaling. Upon illumination, activated BphP1 binds to PpsR2 and releases the promoter under its repression. UV light is harmful to cells while near-infrared signals can penetrate tissues (Chen et al., 2018), which enables applications of the BphP1/PpsR2 system in mammalian cells (Kaberniuk et al., 2016; Redchuk et al., 2017, 2018b).

The two-component systems are similar in signaling and spectrally isolated, enabling construction of multiplexed platforms for multichromatic control of cell behaviors (Tabor et al., 2011; Motta-Mena et al., 2014; Redchuk et al., 2018a). However, their signaling processes require two components and additional genes for chromophore synthesis. These take additional capacity of vectors, limiting the number of genes of interests to be put under light control. To solve this problem, optimized versions of red and green light responding TCSs were built (Schmidl et al., 2014; Ong and Tabor, 2018). The TCSs may be intrinsically complex, relatively slow in signal relay and reversal and less portable, which can be complemented by one-component systems.

### One-component systems

One-component light sensors offer direct control over protein activity, without having to undergo transcription. Sensors in this category can be diverse, but well-characterized types in bacterial cells all belong to the blue light responding LOV family proteins, with a LOV domain fused to different actuators.

YtvA from *B. subtilis* regulates the transcription factor σB for stress response. It consists of a N-terminal LOV domain and a C-terminal Sulfate transporter and anti-sigma factor antagonist (STAS) domain (Ávila-Pérez et al., 2006). Blue light activates the STAT domain which in turn activates the sigma factor and thus activate transcription (Gaidenko et al., 2006; Möglich and Moffat, 2007; Avilapérez et al., 2009). This system is not portable but its LOV domain has been used to design the chimeric YF1/FixJ two-component system (Möglich et al., 2009).

**Figure 2:**
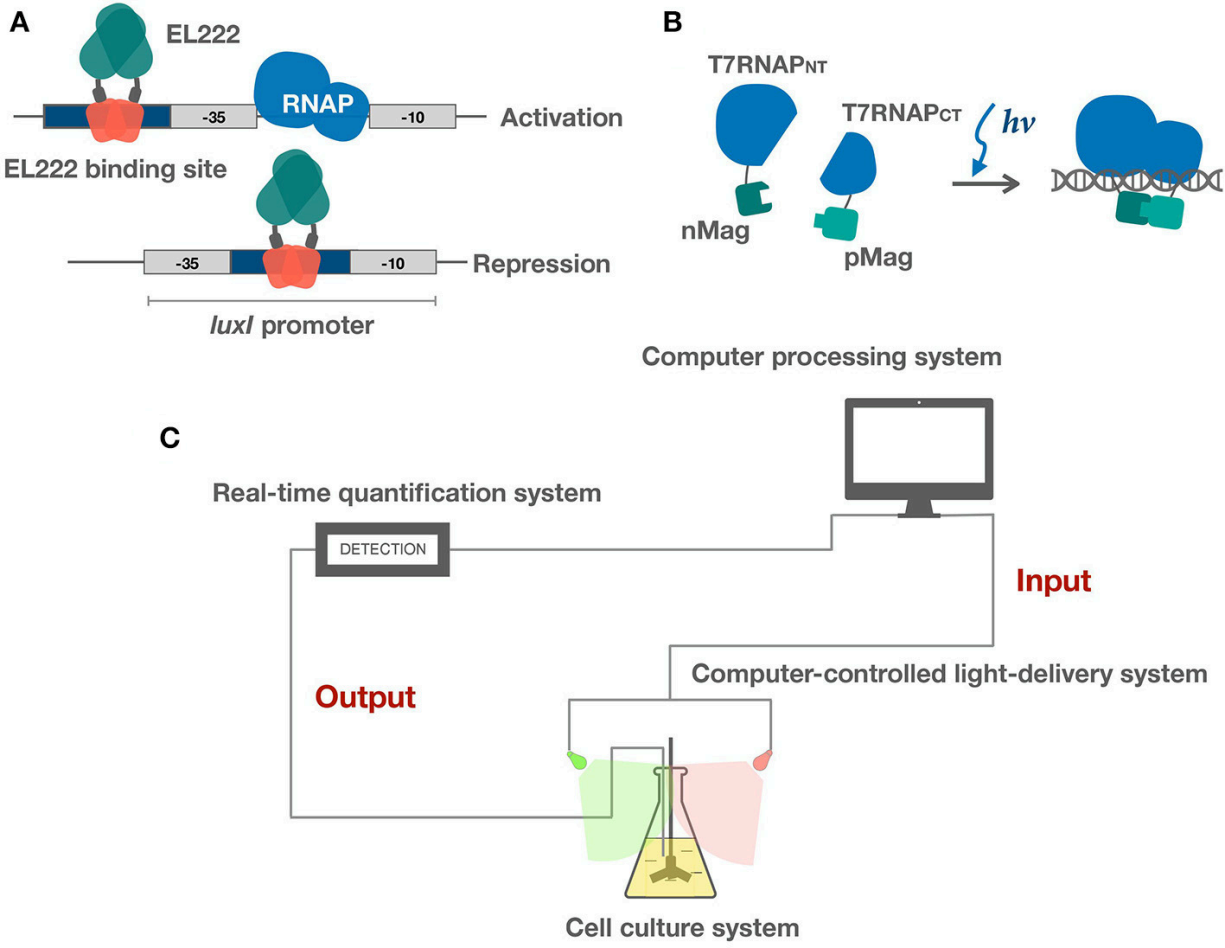
Applications of light sensors in bacteria. **(A)** EL222 light-controlled bidirectional transcription system activates and represses gene expression via different binding strategies. **(B)** Split T7RNAP is brought together by Magnets, reconstituting its transcriptional activity. **(C)** Schematic of microbe-hardware interface and real-time feedback control system. Realtime feedback control is realized by algorithms and hardware. Hardware includes three modules: (1) a cell culture system (2) a real-time quantification system (3) a computer-controlled light-delivery system.

EL222 is a transcription factor from marine bacterium *Erythrobacter litoralis*. It has a LOV domain fused to a HTH DNA binding domain (Zoltowski et al., 2013). The HTH domain is bound and thereby inhibited by the LOV domain in the dark. Upon blue light illumination, the HTH domain is released, thereby enabling DNA-binding (Figure 2A) (Nash et al., 2011; Rivera-Cancel et al., 2015).

VVD (Vivid) from *Neurospora crassa* is another LOV domain-based photoreceptor, forming homodimers in response to blue light (Figure 1C) (Zoltowski et al., 2007, 2009; Zoltowski and Crane, 2008). VVD is unique among all photoreceptors in that its dimer forming capabilities enables direct control over protein activity and localization, thereby enabling control over genome editing, transcription, and beyond. In contrast, EL222 and the TCSs have to go through transcription and are intrinsically inferior regarding of response time and reversibility. Due to its versatility, VVD has been fused to a series of proteins in mammalian cells in controlling transcription (Wang et al., 2012), metal binding (Aper and Merkx, 2016), and Receptor tyrosine kinases (RTK) based cell signaling (Grusch et al., 2014). In bacterial cells, similar versatility is achieved by Magnets, an improved version of VVD, as reviewed in the following. VVD may be susceptible to efficiency and selectivity problems due to its homodimerization property. Magnets addressed this limitation by forming heterodimers based on electrostatic interactions. The designed pairs of photoswitches are thus referred to as positive Magnet (pMag) and negative Magnet (nMag). In addition, the switch-off properties of magnets were also tuned to achieve rapid reversal when light is off (Kawano et al., 2015). Further study has combined pMag with AD (Assembly domains) for increased avidity (Furuya et al., 2017). Two major strategies have been developed to achieve Magnets based light regulations. The first one fuses pMag and nMag to split proteins or dimer forming proteins to offer direct control over protein activities (Nihongaki et al., 2017). The second strategy controls protein activity by tuning its localization. pMag and nMag are fused to different proteins as “bait” and “prey.” Blue light mediates protein colocalization and thus tuning downstream reactions (Yu et al., 2016; Shi et al., 2017; Benedetti et al., 2018).

The one-component systems have miniaturized sizes, easy portability, fast reversibility, and more control sites over the TCSs. But to date, only blue light activated OCSs have been well-characterized in bacteria. Compared with the full spectral programming viability of TCSs, more channels of OCSs should be included.

### Other systems in minority

In addition to the well-tested two-component and one-component systems above, there are also other sensors in minority and underexplored in bacteria. The Cryptochrome 2 (CRY2) and Calcium and integrin binding 1 (CIB1) protein pair is isolated from the plant *Arabidopsis thaliana*. This protein pair is a blue light-controlled dimerization module with no requirement of any exogenously added cofactor, moreover the CRY2 can also oligomerization independently (Figure 1D). The basic helix-loop-helix protein CIB1 binds to the blue light photosensor CRY2 upon light illumination (Repina et al., 2017). The PhyB/PIF (Phytochrome B/Phytochrome interacting factor) system is also from *A. thaliana* and has been harnessed for optogenetic control of protein–protein interactions, mostly in eukaryotes. PhyB consists of a N-terminal photosensory domain and a C-terminal effector domain which binds to transcription factor PIF in response to red light (Repina et al., 2017). Dronpa145N, a mutant of fluorescent protein Dronpa, is switched on by violet light (~400 nm), forming tetramers and is switched off by less energetic cyan light (~500 nm), dissociating into monomers (Figure 1E) (Zhou et al., 2012; Lv et al., 2017). Its oligomerization property has enabled direct control over protein activities. In addition, there are also Cobalamin-binding domains for green signal (Kainrath et al., 2017; Wang et al., 2017) and UV receptor UVRB (Müller et al., 2013). Introduction and validation of these systems may potentially expand the toolbox.

## Light in tuning synthetic circuits

Synthetic biological circuits can be controlled at multiple levels according to the central dogma (Nielsen et al., 2013). Recent advances have harnessed light's ability to control biological circuits at the level of transcription and protein activity. Both two-component and one-component systems have been utilized to replace chemical inducers to achieve rapid activation and deactivation kinetics. Here, we review advances in tuning transcription and protein activity with light and offer a combined introduction of other minor innovations enabled by light's great flexibility.

### Illuminating transcription

Tuning synthetic circuits by tuning transcription is the most common strategy and both two-component and one-component light sensors have been utilized (Table 1). Two-component systems are intrinsically designed for transcriptional control. The red light responding Cph8/OmpR system has been combined with quorum sensing to build a synthetic edge detection program (Tabor et al., 2009). The pDawn and pDusk system demonstrated potential modularity of light sensors in synthetic biology. The YF1/FixJ system is inactivated by blue light, thereby leading to the pDawn system. To achieve blue light activated gene expression, the lamda repressor cI was used as an inverter (Ohlendorf et al., 2012). The pDawn and pDusk system was engineered on one plasmid and does not require additional genes for chromophore synthesis, thereby achieving easier portability (Farzadfard and Lu, 2011; Magaraci et al., 2014). In one practice, the pDawn system has been used to control the expression of an adhesion gene Ag43 and in turn regulates biofilm formation (Jin and Riedelkruse, 2018). In addition, the Cph8/OmpR (Lee et al., 2013), CcaS/CcaR (Nakajima et al., 2016), and YF1/FixJ (Chang et al., 2017) systems were all proposed to control bacterial cell factories by light-mediated transcriptional control (Figure 1A). The TCSs also serve as a useful tool in metabolism engineering of cyanobacteria. As chemical inducers are not ideal considering of large-scale cultivation, the light-regulated TCSs can serve as a good alternative (Song and Park, 2011; Abe et al., 2014). Considering of their modularity and multiple light channels, the two-component systems can be multiplexed to achieve multichromatic control of gene expression (Tabor et al., 2011; Fernandez-Rodriguez et al., 2017). In one recent study, all three RGB channels are engineered in *E. coli* with minimal crosstalk (Fernandez-Rodriguez et al., 2017). A resource allocator based on a highly fragmented T7 RNAP was used in this case to bridge three light signal inputs and transcriptional outputs. Similarly, An iGEM team proposed a prototype of bacterial 3D printer by immobilizing bacteria in gels and using intersection of laser beams to trigger gene expression (Paris-Bettencourt, 2017). Their full spectrum programming ability and similarities in architecture have established the TCSs as a popular choice in light-induced transcriptional control.

A variety of new parts based on one-component systems have been engineered in the past few years. These systems have shown promise in more rapid induction and reversal kinetics and are intrinsically less burdensome and more portable compared to the established two-component systems. A light controlled bidirectional promoter system was built based on EL222 (Jayaraman et al., 2016). Light activated DNA binding features of EL222 has been clearly elucidated. Both light activated and repressed gene expression was achieved by putting the El222 recognition sequence into different regions of the promoter. To achieve light activation, the luxR binding region of the luxI promoter was replaced by the EL222 binding sequence overlapping the −35 region. As the activation mechanism of EL222 is similar to that of the luxR class of DNA binding proteins, light illumination will thus promote EL222 binding and thereby recruiting the RNA polymerase. Similarly, light repressed transcription was achieved by putting the EL222 binding region between the −35 and −10 region and thereby inhibits binding of RNA polymerase (Figure 2A). They also demonstrated that these two promoter systems could function in parallel. An *E. coli* light bulb was made which only gave off bioluminescence in the dark based on an EL222 bistable switch (UCL, 2017). The EL222 system has also recently been introduced into yeast to perform light regulated metabolite production (Zhao et al., 2018). In addition, a recent study has further explored EL222's potential in cell-free optogenetics (Jayaraman et al., 2018). Another class of one-component based transcriptional system was built by fusing split T7 RNAP to VVD and Magnets (Han et al., 2016; Baumschlager et al., 2017). Upon blue light activation, split T7 RNAP was brought together by VVD or Magnets, reconstituting its activity (Figure 2B). This system demonstrates good portability. Only transformation of two genes are required to achieve light induction of gene expression and engineered expression vectors do not have to go through reconstruction due to the use of T7 promoter. Both EL222 and split T7 RNAP systems demonstrate fast reversibility, enabling dynamic control of gene expression. The one-component system shows great potential in tuning transcription although not many parts have been engineered. The most significant advantage of sensors within this category is that the light sensory parts are miniaturized, thereby leaving more capacity for targeted genes of interests. In addition, less signaling and reversal time is required due to the one-component architecture, thereby enabling precise dynamic controls.

Light could also be combined with other signals to build logic gates and complex layered circuits (Camsund et al., 2011; Drepper et al., 2011; Gardner and Deiters, 2012). One of the strategies is to combine light and other signals with the CRISPR technology, which has been proved to achieve numerous functions (Nielsen and Voigt, 2014). Specifically, a gRNA could be put under light controlled promoters to combine light with CRISPR activation and deactivation of downstream gene expression (Bikard et al., 2013; Qi Lei et al., 2013; Jusiak et al., 2016). This enables light control over genes on the chromosome. Such a principle has been practiced in metabolism engineering, where gRNAs are transcribed in response to light and then combined with dcas9 for redirection of metabolic flux (Fernandez-Rodriguez et al., 2017). The 2017 HZAU iGEM team proposed to use light to control bacteria cell replication in a similar methodology (HZAU-China, 2017). Instead of transcribing a gRNA upon light illumination, they fused pMag and nMag with split dcas9. Upon light illumination, this binds to DnaA binding sites and inhibit DnaA binding which is essential for DNA and cell replication. Similar methodologies could be used to carry out real-time feedback regulation of cell growth and even constructing microbial consortia with predefined ratios with more light channels available.

### Illuminating protein activities

In addition to transcriptional control, biological circuits can also be tuned by offering direct control over protein activities (Table 1). Optical control of protein activities has been established in eukaryotic cells in part due to the access to a wide range of sensors (Brechun et al., 2017; Liu and Tucker, 2017). These sensors belong to one-component systems which undergo conformational changes or shifts in their dimerization or oligomerization states upon light illumination. Although widely applied in eukaryotic cells, light's potential in regulating protein activities remains underexplored in bacteria and this correlates with the fact that one-component systems have just been explored recently in bacteria. Here, we review recent practices in bacteria cells and offer a very brief introduction of strategies used in eukaryotic systems which could serve as a template for potential counterpart engineering in bacterial cells.

Practices in bacterial cells have only been emerging in recent years. The magnets were used to control biofilms. pMag was displayed on the surface of *E. coli* and nMag was immobilized on a surface, thus achieving blue light control of biofilm formation (Chen and Wegner, 2017). Also, biofilm formation has been controlled by blue light via other strategies (Huang et al., 2018; Jin and Riedelkruse, 2018; Pu et al., 2018). In addition, light has also been used to control protein self-assembly in *E. coli* (Yu et al., 2017). The phytochrome family has sensing ranges spanning the spectrum and the bacteriaphytochromes can sense far red light and have thus been used to construct light controlled adenylate and guanylate cyclases for cell signaling (Ryu and Gomelsky, 2014; Ryu et al., 2014). In addition to the major TCSs and OCSs, highly specialized light controlled proton pumps have been proposed to control bacteria movement, enabling design of micromotors (Walter et al., 2007; Lozano et al., 2016; Vizsnyiczai et al., 2017).

A major topic in eukaryotic cells is tunable protein degradation, which is practically significant in mammalian cell biology for proteins too essential to be depleted. Synthetic light inducible degrons have been engineered in yeast (Usherenko et al., 2014; Lutz et al., 2016) and higher eukaryotes (Renicke et al., 2013). The most common strategy is to fuse a LOV family domain for caging of the degradation tag. In dark, the degradation tag is “caged” by the LOV family protein and is only released and got access to degradation upon light illumination (Figure 1B). Similar systems could be engineered in bacterial cells by simply replacing the eukaryotic degron with a bacterial degradation tag which has been intensively investigated (Cameron and Collins, 2014; Lauritsen et al., 2018).

Light has also been used to control subcellular localizations of proteins (Brechun et al., 2017). One strategy is based on separated light sensors which can be brought together upon illumination, including Phy/PIF and magnets. One part of the system can be anchored to the target sites while the other could be fused to the protein of interest. Upon light induction, the protein of interest will be driven to the target sites by complemented light sensors. In one study, protein localizations to nucleus, endosomes, and cell membrane were achieved by the PhyB/PIF6 system (Yang et al., 2013). Another strategy is to fuse LOV family proteins to cage the signal peptide (Brechun et al., 2017). Different systems have been engineered to control import to and export from the nucleus (Di Ventura and Kuhlman, 2016; Yumerefendi et al., 2016) and a recent study has proposed a strategy to control tridirectional protein localization among the nucleus, cytoplasm, and plasma membrane (Redchuk et al., 2018a). These strategies in eukaryotes may inspire engineering protein subcellular localization in bacterial cells and beyond.

## Light in bridging microbes and hardware

To realize real-time and remote feedback control of cell cultures, a major strategy is to use hardware to monitor culture conditions and deliver signals to alter cell behaviors responsively (Vance et al., 2002; Gardner et al., 2003; Mettetal et al., 2008; Muzzey et al., 2009; Shimizu et al., 2010). However, a huge gap exists between cultured cells and microbes as there lacks a signal with kinetics rapid enough for feedback control. Light as a new signal has successfully filled in this gap between cultured microbes and the hardware for its unique switch-on and switch-off kinetics (Gerhardt et al., 2016). In previous studies light has been used to construct oscillators (Jayaraman et al., 2016) and dynamically regulate gene expressions (Milias-Argeitis et al., 2011, 2016; Melendez et al., 2014; Olson and Tabor, 2014; Olson et al., 2014) and protein localizations (Toettcher et al., 2011).

Feedback control in these studies have been realized by the coordination of hardware and algorithms. The hardware parts are firstly responsible for the cultivation of microbes and real-time characterization of growth conditions. These data enter the computer, and algorithms help determine the specific manipulations which are sent to the hardware to deliver light to the cells, forming a close loop (Figure 2C). Below we offer an introduction to the hardware and algorithms and present a brief outlook for potentials of light-based feedback control.

Hardware shares an essential three-part architecture: (a) an adapted cell culture system; (b) a real-time quantification system; and (c) a computer-controlled light delivery system (Figure 2C) (Milias-Argeitis et al., 2016; Hennemann et al., 2018; Mahajan and Rai, 2018). **The cell culture system** is supposed to be designed according to experimental purposes. It tends to involve an integrated heated magnetic stirrer both for maintaining temperature and aeration conditions and ensuring random sampling. **The quantification system** tracks cell properties and medium conditions in real time. Common cell properties include OD, intensity of fluorescence, or luminescence while medium conditions may include pH level, concentrations of organic, and inorganic molecules. Microfluidic devices are often used in practice. **The light delivery system** is a controlled light source with tunable wavelength and intensity. The wavelength is mainly determined by signaling properties of the light sensors encoded *in vivo* while intensity and duration of illumination are determined dynamically according to data input from the quantification system and previous modeling data.

Current algorithms fall into two categories: the proportional-integral (PI) algorithm (Dorf and Bishop, 2011) and The model predictive control (MPC) algorithm (Camacho and Alba, 2013). The PI algorithm is the most popular variation of the proportional-integral-derivative (PID) algorithm. It continuously accepts the error between a desired setpoint (SP) and a measured process variable (PV) to form the sum of two terms: one proportional to the current error, and the other proportional to the time integral of the error. Using the result, the PI algorithm applies responsive correction to the next control function. However, PI controller are not capable to accurately tracking time-varying references, unless they change very slowly. The MPC algorithm requires a model of the controlled system, which is different from the PI algorithm. Based on the model, it uses the current dynamic state of a process to predict future values of the outputs, and then the appropriate changes in the input variables can be calculated based on both predictions and the current state. Thus, the accurate model predictions can provide early warnings of potential problems. However, requirement of an established model restricts its application, especially for complex cellular behaviors where a model is difficult to construct (Milias-Argeitis et al., 2016).

With the development of hardware and algorithms, optogenetic feedback control is exploring the potential in other prospects including both basic science and engineering. For example, it can be used to analyze other dynamic processes such as cell cycles (McAdams and Shapiro, 2003), differentiation (Kuchina et al., 2011; Ray et al., 2011; Levine et al., 2012; Vishnoi et al., 2013), stress responses (Young et al., 2013), and migration (Weitzman and Hahn, 2014). Besides, optogenetic feedback control can be employed to optimize metabolic pathways, especially when metabolites can disturb normal cell behaviors (Zaslaver et al., 2004; Temme et al., 2012). Also, the capability of optogenetic feedback control can be further explored with the development of novel quantification and measurement strategies. For example, employing RNA sequencing for measuring mRNA in real time can enable more parameter readouts and meanwhile enhance accuracy compared to the fluorescent protein approach (Olson and Tabor, 2014).

## Concluding remark

Controlling cellular behaviors have been a significant topic in synthetic biology. Light has demonstrated its great capacity in controlling cellular behaviors for its minimal toxicity and rapid activation and deactivation kinetics compared to chemical inducers. While mammalian optogenetics have been established for years, programming bacteria with light has been an emerging new field. In the past 15 years, a variety of Two-component and One-component systems have been engineered. These systems have been widely utilized in tuning synthetic circuits at the level of transcription and protein activity. Given the existing sensor toolbox and accumulated practices at the level of transcriptional and protein activity control, light has also been further explored to bridge the gap between cultured microbes and hardwares. Real-time feedback control over cell behaviors was achieved thanks to light's unique signaling properties.

Despite the great promise, practices have been mainly restricted by limited choices of light sensors. The TCSs demonstrate potential in multiplexed light programming but takes up additional space on vectors and are inferior in reversal kinetics. On the other hand, the OCSs are miniaturized and rapidly switched off but limited to blue light. This leads to the necessity to expand the toolbox: The first strategy is to learn from established mammalian optogenetics, introducing new systems into bacteria cells; A second strategy is to continually mine naturally existing systems, the same as how some current systems were discovered; and a third strategy is to improve current parts by modification and construct new parts based on their modularity, which has demonstrated success in the development of most OCSs.

Programming bacteria behaviors with light has shown great promise thanks to light's unique signaling properties. With current sensor systems improved and new systems to be explored, we believe light will illuminate more bacteria behaviors in the near future.

## Author contributions

ZL wrote the manuscript with the assistance from JZ (part of the manuscript), JJ (Figures 1, 2, and part of the manuscript), and ZG (Table and part of the manuscript). QL and QQ conceptualized the idea and objectives of the review.

### Conflict of interest statement

The authors declare that the research was conducted in the absence of any commercial or financial relationships that could be construed as a potential conflict of interest.
